# Lycorine Ameliorates Thioacetamide-Induced Hepatic Fibrosis in Rats: Emphasis on Antioxidant, Anti-Inflammatory, and STAT3 Inhibition Effects

**DOI:** 10.3390/ph15030369

**Published:** 2022-03-18

**Authors:** Huda Mohammed Alkreathy, Ahmed Esmat

**Affiliations:** 1 Department of Pharmacology, Faculty of Medicine, King Abdulaziz University, Jeddah 21589, Saudi Arabia; halkreathy@kau.edu.sa; 2 Department of Pharmacology and Toxicology, Faculty of Pharmacy, Ain Shams University, Cairo 11566, Egypt

**Keywords:** lycorine, thioacetamide, liver fibrosis, STAT3

## Abstract

Liver fibrosis is a foremost medical concern worldwide. In Saudi Arabia, numerous risk factors contribute to its high rates. Lycorine—a natural alkaloid—has antioxidant, anti-inflammatory, and antitumor activates. It has been reported to inhibit STAT3 in cancer. Therefore, this study aimed at investigating the possible antifibrotic effect of lycorine against thioacetamide (TAA)-induced liver fibrosis in rats and at elucidating the possible mechanisms. Liver fibrosis was induced by TAA (200 mg/kg i.p.), three per week for four weeks. Treatment with lycorine (0.5 and 1 mg/kg/d) amended TAA-induced rise of serum transaminases that was confirmed histopathologically. Moreover, it ameliorated liver fibrosis in a dose-dependent manner, as indicated by hindering the TAA-induced increase of hepatic hydroxyproline content, α-smooth muscle actin (α-SMA) and transforming growth factor (TGF-β1) expressions. TAA-induced oxidative stress was amended by lycorine treatment via restoring reduced glutathione and diminishing lipid peroxidation. Moreover, lycorine ameliorated hepatic inflammation by preventing the rise of inflammatory cytokines. Notably, lycorine inhibited STAT3 activity, as evidenced by the decreased phospho-STAT3 expression, accompanied by the elevation of the hepatic Bax/Bcl-2 ratio. In conclusion, lycorine hinders TAA-induced liver fibrosis in rats, due to—at least partly—its antioxidative and anti-inflammatory properties, along with its ability to inhibit STAT3 signaling.

## 1. Introduction

Liver damage could lead to deleterious consequences to the human body, including death [1]. Liver disease is responsible for two million deaths every year globally, with cirrhosis representing the 11th most common cause of mortality worldwide [2]. Metabolic disorders, including insulin resistance, diabetes mellitus, obesity, and dyslipidemia, contribute to liver damage [3]. Liver fibrosis is a healing process where extracellular matrix (ECM) proteins accumulate as a substituent of hepatocytes after persistent liver injury [4,5]. Both oxidative stress and inflammation were conveyed in all fibrotic disorders with chronic damage and remodeling [6]. Interestingly, Signal Transducer and Activator of Transcription factor (STAT3) is physiologically stimulated during tissue repair as a result of inflammation, participating in the initial steps of the healing process [7]. However, if it is insistently activated, it could contribute to damaging effects and various disorders, including organ fibrosis [8,9]. STAT3 has been well-known to confer potent proliferative actions and apoptotic resistance, which could contribute to the replication of myofibroblasts, with a subsequent buildup of connective tissue in fibrotic states [10]. Therefore, blocking STAT3 could be a favorable aim for antifibrotic action.

Inopportunely, there is no reasonable therapy for hepatic fibrosis, which requires chronic use of safe and effective drugs [3]. On the other hand, respectable recognition of natural products has been increasing among the scientific and public communities [11]. In this regard, lycorine—a natural alkaloid extracted from the genus Amaryllidaceae—was formerly reported to inhibit the growth and cell division in yeasts, algae, and higher plants [12]. Then, studies were continued to detect other activities of lycorine, including antitumor, anti-inflammatory, and antioxidant effects [13,14,15]. Another study [16] indicated the hepatoprotecive effects of lycorine in an animal model of CCl_4_-indced acute hepatotoxicity. More recently, lycorine has been reported to activate the mitochondrial apoptosis pathway via targeting STAT3, which was effective at inhibiting the malignant growth of colorectal cancer both in-vivo and in-vitro [17]. Although the antifibrotic activity of lycorine has been investigated in experimental models like bleomycin-induced pulmonary fibrosis [18], as well as several experimentally-induced cardiac dysfunctions [19,20,21], there is a paucity of information regarding the antifibrotic activity of lycorine against experimentally-induced hepatic fibrosis with respect to STAT3 activity. Thioacetamide (TAA) was utilized in the current study because it induces liver fibrosis similar to human alcoholic fibrogenesis, and results in a low animal mortality [22]. Therefore, this study aimed at investigating the possible antifibrotic effect of lycorine against thioacetamide (TAA)-induced liver fibrosis in rats and at elucidating the possible mechanisms.

## 2. Results

### 2.1. Assessment of Hepatic Fibrosis

**Figure 1:**
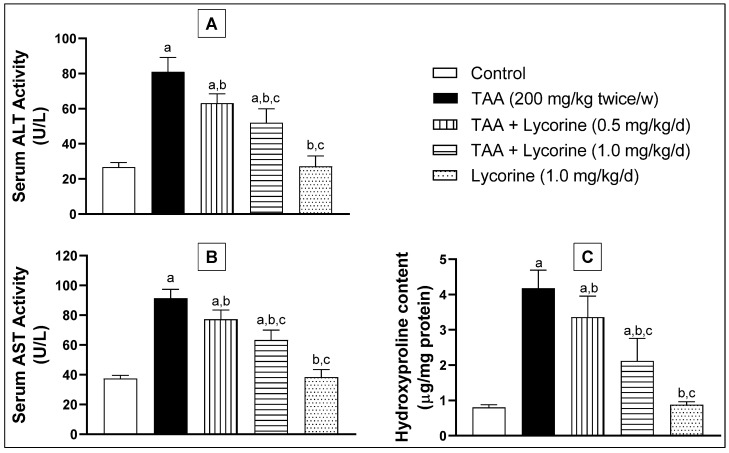
Effect of lycorine on (**A**) serum ALT activity, (**B**) serum AST activity, and (**C**) hepatic hydroxyproline in a rat model of TAA-induced liver fibrosis. Data are displayed as mean ± S.D. *n* = 6. Statistical analysis was carried out by one-way ANOVA followed by Tukey’s post-*hoc* test. (a) Statistical significance from the corresponding control at *p* < 0.05. (b) Statistical significance from TAA-challenged group at *p* < 0.05. (c) Statistical significance from the TAA + lycorine 0.5 mg/kg/d group at *p* < 0.05. TAA: thioacetamide; ALT: alanine transaminase; AST: aspartate transaminase.

Upon challenging with TAA, intracellular hepatic enzymes ALT and AST were seeped into the blood, as shown in Figure 1. This effect was demonstrated by significant rises of ALT and AST serum activities by about 3- and 2.4-folds, respectively, compared to the corresponding control rats. Furthermore, TAA caused a significant (5-fold) surge in the fibrosis biomarker “hydroxyproline” in hepatic tissue homogenate, compared to the control group. Treatment with lycorine (0.5 and 1 mg/kg/d) significantly reduced ALT activity by about 22% and 36%, respectively, compared to the TAA-challenged group. A similar pattern of activity was observed with the AST enzymatic activity. Regarding the hydroxyproline content, lycorine treatment (0.5 and 1 mg/kg/d) was able to decrease it by about 19% and 49%, respectively, in relation to the TAA-exposed group. Notably, the lycorine-alone treated group showed no significant difference from the corresponding control groups for all of the assessed biomarkers.

**Figure 2:**
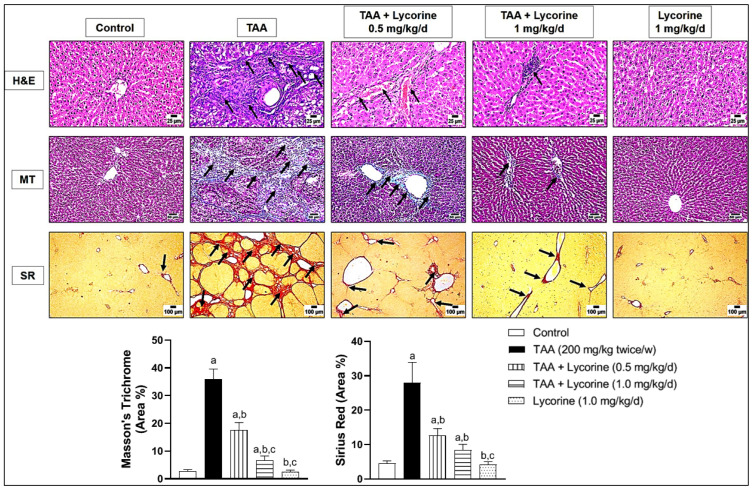
Histopathological examination of liver sections stained by hematoxylin and eosin (H&E), Masson’s Trichrome (MT) and Sirius Red (SR) in a rat model of thioacetamide (TAA)-induced liver fibrosis. The control group shows a normal lobular architecture (H&E), accompanied by a normal distribution of fine collagen fibers around the central vein (MT and SR); the TAA-exposed group displays thick fibrous strands that surrounds the hepatic lobules with mononuclear inflammatory cell infiltration (arrows) and hepatocellular vacuolar degeneration (H&E), accompanied by abundant fibroplasia completely surrounding the hepatic lobules (blue with MT and red with SR); treatment with lycorine (0.5 mg/kg/d) shows a moderate improvement of the histopathological changes by all stains; treatment with lycorine (1 mg/kg/d) shows a marked improvement of histopathological changes, except for minor inflammatory cells infiltration (H&E) together with fine collagen deposition (MT & SR), as indicated by arrows; the lycorine-alone group (1 mg/kg/d) displays apparently normal hepatic tissue (H&E) and collagen deposition (MT and SR). MT and SR staining was quantified as (area %). Data are presented as mean ± S.D. (*n* = 6). (a), (b), (c) Statistically significant from the control, TAA, and TAA + lycorine 0.5 mg/kg/d groups, respectively, at *p* < 0.05 using one-way analysis of variance (ANOVA) followed by Tukey’s as a post-*hoc* test.

These biochemical findings were further substantiated by histopathological examination of the representative liver tissues using three different staining—H&E, MT, and SR. As shown in Figure 2, H&E staining of the control sections revealed a normal histologic structure, with hepatocytes arranged in a regular lobular architecture. This was accompanied by normal distribution of fine collagen fibers around the central vein, as shown in MT & SR staining. On the other hand, challenging with TAA resulted in excessive hepatic fibrosis. Staining with H&E revealed thick fibrous strands resulting in complete bridging fibrosis that surrounded the hepatic lobules with mononuclear inflammatory cell infiltration. The hepatocytes suffered from vacuolar degeneration and necrosis due to diffuse extensive fibrosis. These pathological changes were further confirmed by abundant fibroplasia, stained blue with MT and red with SR stains, replacing the hepatic parenchyma and completely surrounding the hepatic lobules. Treatment with lycorine (0.5 and 1 mg/kg/d) showed a dose-related improvement of histopathological changes (H&E) and collagen deposition (MT & SR) in the examined liver sections. The lycorine-alone group showed apparently normal hepatic tissue and collagen deposition.

**Figure 3:**
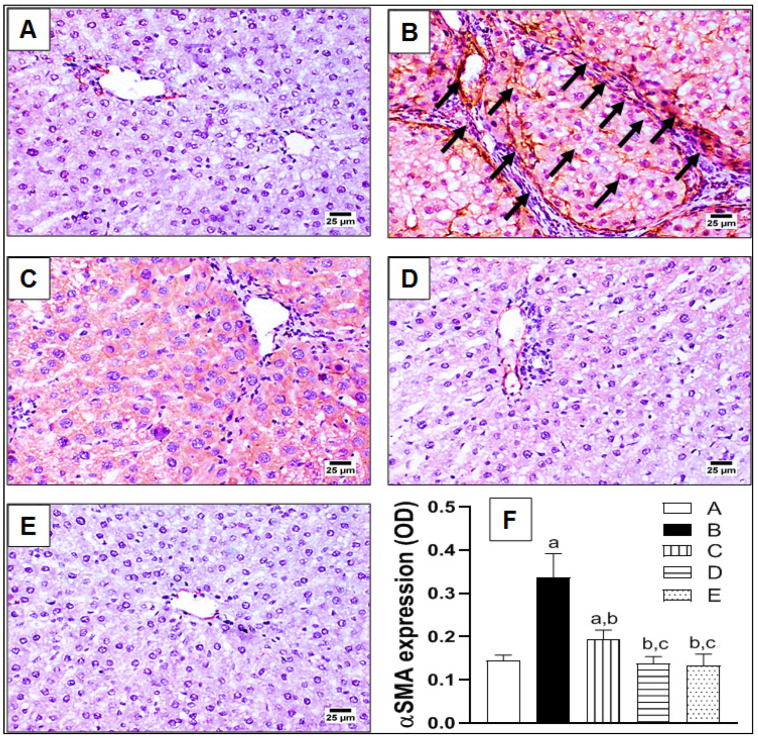
The effect of lycorine on expression of α smooth muscle actin (αSMA) by immunohistochemical staining in a rat model of TAA-induced liver fibrosis. (**A**) Control group; (**B**) TAA-challenged group; (**C**,**D**) lycorine-treated groups at doses 0.5 and 1.0 mg/kg, respectively; (**E**) lycorine-alone group; (**F**) quantitative image analysis for αSMA immunohistochemical staining, as optical density (OD) across six different fields. Data are presented as mean ± S.D. (*n* = 6). (a), (b), (c) Statistically significant from the control, TAA, and TAA + lycorine 0.5 mg/kg/d groups, respectively, at *p* < 0.05 using one-way analysis of variance (ANOVA) followed by Tukey’s as a post-*hoc* test.

**Figure 4:**
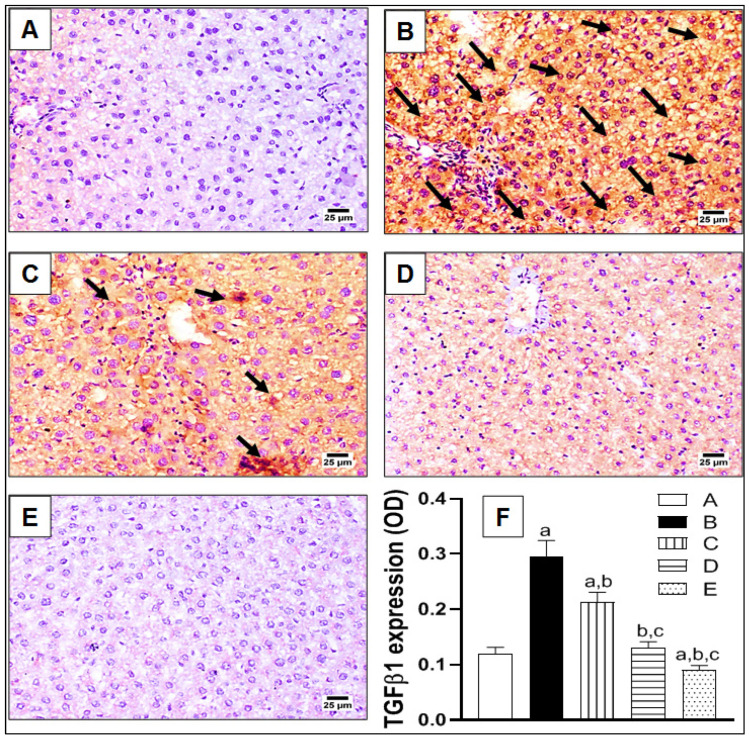
The effect of lycorine on the expression of transforming growth factor β1 (TGFβ1) by immunohistochemical staining in a rat model of TAA-induced liver fibrosis. (**A**) Control group; (**B**) TAA-challenged group; (**C**,**D**) lycorine-treated groups at doses of 0.5 and 1.0 mg/kg, respectively; (**E**) lycorine-alone group; (**F**): quantitative image analysis for TGFβ immunohistochemical staining, as optical density (OD) across six different fields. Data are presented as mean ± S.D. (*n* = 6). (a), (b), (c) Statistically significant from the control, TAA, and TAA + lycorine 0.5 mg/kg/d groups, respectively, at *p* < 0.05 using one-way analysis of variance (ANOVA) followed by Tukey’s as a post-*hoc* test.

To further confirm hepatic fibrosis on a molecular level, the expressions of fibrotic biomarkers αSMA and TGFβ1 were assessed immunohistochemically, as indicated in Figure 3 and Figure 4, respectively. In brief, the control group showed a restricted expression of αSMA around the wall of the hepatic arteries and portal veins in the portal areas (Figure 3A). Meanwhile, excessive expression of αSMA was noted in the TAA-challenged group, with abundant fibrotic tissue extending from the portal areas and surrounding the hepatic lobules, as indicated by arrows (Figure 3B). In fact, this correlates with the previously-illustrated staining pattern of hepatic sections by MT and SR (Figure 2). Treatment with lycorine (0.5 and 1 mg/kg/d) displayed an obvious reduction of αSMA expression in a dose-related manner (Figure 3C,D, respectively). Finally, the lycorine-alone group revealed a normal expression of αSMA in the hepatic architecture (Figure 3E). Statistical analysis of the αSMA expression as optical density (OD) showed a significant (>2-fold) increase of OD in the TAA-exposed group when compared to the control, as shown in Figure 3F. Interestingly, the absence of a significant difference in OD was observed when comparing lycorine (1 mg/kg/d) either alone or in the treated animals with the control value. A similar pattern of expression was detected with TGFβ1 immunohistochemical staining, as displayed in Figure 4. Yet, TGFβ1 expression involved the whole hepatic section and was not only restricted to portal areas and around hepatic lobules, as indicated by arrows (Figure 4B). Again, lycorine treatment induced a dose-dependent decline of TGFβ1 expression (Figure 4C,D). These observations were further confirmed by quantitation as OD (Figure 4F). It is noted that lycorine alone was capable of significantly reducing the baseline TGFβ expression of the control value.

### 2.2. Assessment of Oxidative Status

**Figure 5:**
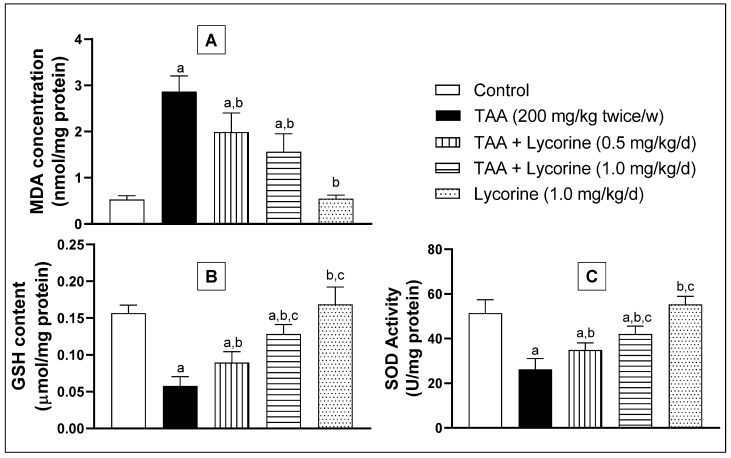
Effect of lycorine on oxidative markers. (**A**) MDA concentration, (**B**) GSH content, and (**C**) SOD activity in a rat model of TAA-induced liver fibrosis. Data are displayed as mean ± S.D. (*n* = 6). Statistical analysis was carried out by one-way ANOVA followed by Tukey’s post-*hoc* test. (a) Statistical significance from the corresponding control at *p* < 0.05. (b) Statistical significance from the TAA-challenged group at *p* < 0.05. (c) Statistical significance from the TAA + lycorine 0.5 mg/kg/d group at *p* < 0.05. TAA: thioacetamide; MDA: malonedialdhyde; GSH: reduced glutathione, SOD: superoxide dismutase.

Hepatic oxidative status was investigated via assessing the homogenate MDA concentration (a lipid peroxidation marker), GSH content (an antioxidant tripeptide), and SOD antioxidant enzyme activity. As indicated in Figure 5, exposure to TAA induced oxidative stress, as manifested by a significant increase in MDA concentration by more than five-fold, compared to the control. Meanwhile, TAA abolished the GSH content and significantly reduced the SOD enzymatic activity by about 74% and 50%, respectively, related to the corresponding control groups. On the other hand, treatment with lycorine (0.5 and 1 mg/kg/d) obviously improved the oxidative status in a dose-dependent way, being able to significantly reduce the MDA concentration, replenish GSH content, and boost SOD enzymatic activity in hepatic homogenate, compared to the matching control values. In addition, lycorine alone did not induce any significant difference in the assessed oxidative biomarkers from the corresponding control groups.

### 2.3. Assessment of Inflammatory Status

**Figure 6:**
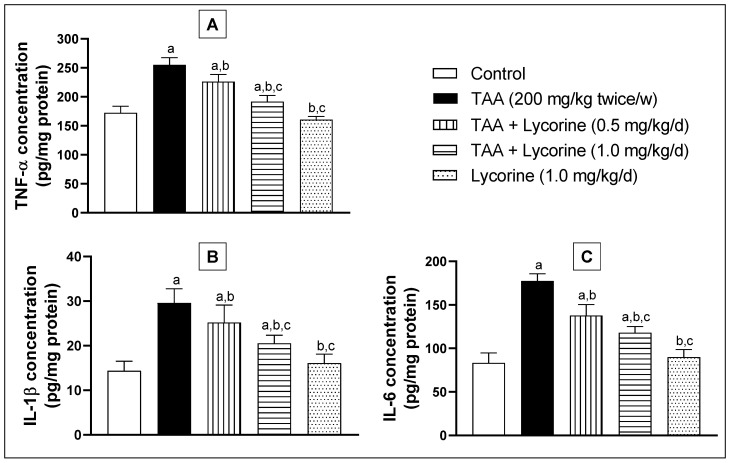
Effect of lycorine on the homogenate concentration of (**A**) TNF-α, (**B**) IL-1β, and (**C**) IL-6 in a rat model of TAA-induced liver fibrosis. Data are presented as mean ± S.D. (*n* = 6). Statistical analysis was carried out by one-way ANOVA followed by Tukey’s post-*hoc* test. (a) Statistical significance from the corresponding control at *p* < 0.05. (b) Statistical significance from the TAA-challenged group at *p* < 0.05. (c) Statistical significance from the TAA + lycorine 0.5 mg/kg/d group at *p* < 0.05. TAA: thioacetamide; TNF-α: tumor necrosis factor-alpha; IL-1β: interleukin-1beta; IL-6: interleukin-6.

Hepatic inflammation was evaluated via assessing inflammatory biomarkers TNFα, IL-1β, and IL-6 in the tissue homogenate using ELISA kits, as indicated in Figure 6. Animals challenged with TAA showed a significant increase of TNFα concentration in their hepatic tissue homogenates by about 50% in comparison to the control group (Figure 6A). Lycorine treatment caused significant declines of TNFα concentration by 11% and 25% at the tested doses (0.5 and 1 mg/kg/d, respectively), in relation to the TAA-exposed group. Moreover, assessment of the IL-1β and IL-6 hepatic concentrations displayed a comparable pattern, where TAA resulted in >2-fold increased concentrations of both biomarkers, as shown in Figure 6B,C. Once more, lycorine treatment ameliorated the TAA-induced elevation of IL-1β and IL-6 hepatic concentrations in a dose-dependent manner. Notably, the lycorine-alone group failed to show any significant differences in TNFα, IL-1β, and IL-6 hepatic concentrations from their corresponding controls.

### 2.4. Assessment of STAT3

**Figure 7:**
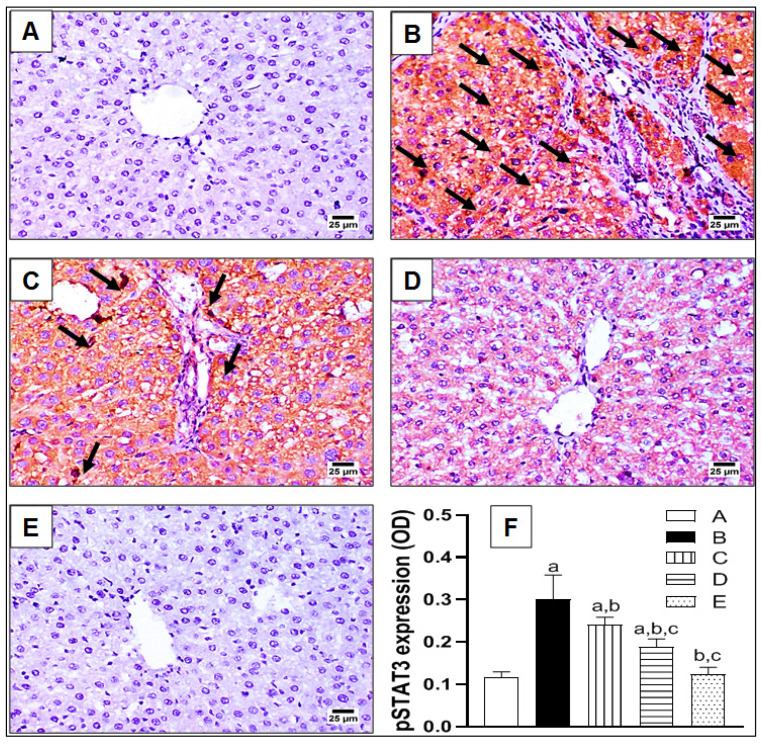
The effect of lycorine on the expression of phospho-STAT3 (pSTAT3) by immunohistochemical staining in a rat model of TAA-induced liver fibrosis. (**A**) Control group; (**B**): TAA-challenged group; (**C**,**D**) lycorine-treated groups at doses 0.5 and 1.0 mg/kg, respectively; (**E**) lycorine-alone group; (**F**) quantitative image analysis for pSTAT3 immunohistochemical staining, as optical density (OD) across 6 different fields. Data are presented as mean ± S.D. (*n* = 6). (a), (b), (c) Statistically significant from the control, TAA, and TAA + lycorine 0.5 mg/kg/d groups, respectively, at *p* < 0.05 using one-way analysis of variance (ANOVA) followed by Tukey’s as a post-*hoc* test.

As indicted in Figure 7, the expression of pSTAT3—the active form of STAT3—was investigated immunohistochemically in hepatic tissue sections following TAA-induced hepatic fibrosis. The control animals showed a very limited expression of pSTAT3 (Figure 7A). On the contrary, it was excessively expressed upon TAA challenge, manifested by arrows and brown coloration, as shown in Figure 7B. Treatment with lycorine caused a dose-related decline in pSTAT3 expression, compared to the TAA-group (Figure 7C,D). In order to evaluate these differences quantitatively, the pSTAT3 expression was statistically analyzed as OD (Figure 7E), where TAA exposure resulted in a three-fold rise of pSTAT3 expression in comparison to the control group. However, this higher expression was significantly ameliorated by about 20% and 37% upon treatment with lycorine at 0.5 and 1 mg/kg/d, respectively.

**Figure 8:**
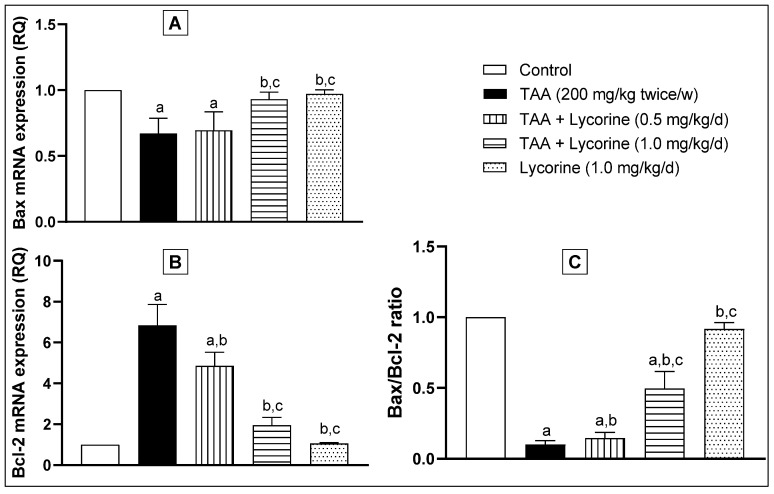
The effect of lycorine on the quantitative RT-PCR of (**A**) Bax and (**B**) Bcl-2 mRNA expression, expressed as relative quantitation (RQ), and the (**C**) Bax/Bcl-2 ratio. Data are presented as mean ± S.D. (*n* = 6). Statistical analysis was carried out by one-way ANOVA followed by Tukey’s post-*hoc* test. (a) Statistical significance from the corresponding control at *p* < 0.05. (b) Statistical significance from the TAA-challenged group at *p* < 0.05. (c) Statistical significance from the TAA + lycorine 0.5 mg/kg/d group at *p* < 0.05.

In this regard, the gene expressions of the proapoptotic “Bax” and antiapoptotic “Bcl-2” proteins were performed as downstream to the STAT3 signaling pathway. In brief, exposure to TAA significantly down-regulated Bax and up-regulated Bcl-2 mRNA expressions (Figure 8A,B, respectively), leading to marked decline in the calculated Bax/Bcl-2 ratio by 90%, compared to the control, as shown in Figure 8C. Treatment with lycorine (0.5 mg/kg/d) caused a trivial but significant elevation of the Bax/Bcl-2 ratio. This effect became more noticeable with a higher dose of lycorine (1 mg/kg/d), which significantly improved the Bax/Bcl-2 ratio to about 50% of its control value. Yet again, the lycorine-alone group showed no significant difference in pSTAT3 protein expression, as well as Bax and Bcl-2 gene expressions, compared to the corresponding control group.

## 3. Discussion

Presently, there is no reasonable remedy for liver fibrosis [23]. Lycorine is a natural alkaloid that possesses antitumor [13,14], antioxidant [24], and anti-inflammatory activities [25], in addition to being hepatoprotective in CCl_4_-induced acute hepatotoxicity [16]. Moreover, it has been reported to activate the mitochondrial apoptosis pathway via targeting STAT3 [17]. Therefore, the current work aimed at investigating the potential antifibrotic effect of lycorine against thioacetamide-induced liver fibrosis in rats, along with elucidating the possible causal mechanisms with respect to the STAT3 pathway.

For decades, TAA has been known for its ability to induce liver fibrosis [26]. Yet, its molecular mechanism is still not fully unstated. Indeed, TAA undergoes bioactivation in the liver via oxidation processes via hepatic CYP2E1 [27,28]. This leads to the formation of reactive metabolites, namely, S-oxide and SS-dioxide, which are apparently responsible for TAA-induced hepatic injury [29]. Basically, liver injury activates hepatic stellate cells (HSCs) to multiply and secrete extracellular matrix (ECM) components like interstitial collagens [30,31]. Moreover, HSCs are converted into myofibroblasts expressing α-SMA, which induces fibrogenesis and tissue stiffness [32]. By mechanisms including autocrine and paracrine pathways, ECM components promote growth factor signaling, principally by TGF-β, which in-turn add to the activation of HSCs, generating a positive feedback circle [33]. It is worth mentioning that the TGF-β family comprises three isoforms TGF-β 1, 2, and 3 with different biological activities [34]. The isoform TGF-β1 gained the most importance due to its pleiotropic nature, extending from tissue fibrosis to preparing the microenvironment for carcinogenesis [35,36]. With regard to hepatic pathogenesis, the assessed TGF-β1 has been reported to have a critical role in hepatic fibrosis through direct activation of HSCs and accumulation of ECM [37]. In the current study, lycorine treatment significantly diminished TAA-induced hepatic injury by significantly ameliorating the rise of hepatic transaminases “ALT and AST” and the fibrotic marker “hydroxyproline”. This was confirmed by its ability to improve histopathological-fibrotic changes, as evidenced by H&E, MT, and SR staining, and to guard against the excessive immunohistochemical expression of αSMA and TGFβ1. These findings are in line with recent studies investigating the antifibrotic activity of lycorine against bleomycin-induced pulmonary fibrosis [18], as well as experimentally-induced cardiac dysfunction [19,20,21].

Oxidative stress and inflammation have crucial roles in HSCs activation, which ultimately ends in fibrogenesis [32]. In the current study, lycorine treatment significantly improved TAA-induced oxidative stress in hepatic tissues, as manifested by the enhanced GSH content, SOD activity, and reduced lipid peroxidation. This is in agreement with previous reports, confirming the antioxidant activity of lycorine as a DPPH scavenger [15] and its capability of protecting human erythrocytes against 2-amidinopropane-induced oxidative hemolysis [24]. In addition, lycorine exhibited hepatoprotective effects against CCl4-induced oxidative stress in mice [38]. Concerning inflammatory cascade, it is notorious that activated HSCs express cytokines that are key mediators of fibrogenesis. Specifically, the proinflammatory cytokine TNF-α is released from HSCs secondary to TGF-β1, which in turn inaugurates inflammatory responses via stimulating the secretion of other proinflammatory cytokines, such as IL-1β [39]. In this, lycorine treatment significantly ameliorated TAA-induced hepatic inflammation, as manifested by diminishing the rise of TNF-α, IL-1β, and IL-6. Notably, lycorine was found to possess an efficient anti-inflammatory activity in numerous experimental models such as rat adjuvant arthritis [40] carrageen-induced rat paw edema [16]; and lipopolysaccharide (LPS) challenge in RAW264.7 cells via inhibiting iNOS, PGE2, TNF-α, IL-6, and JAK-STAT3 signaling pathways [25].

Recently, STAT3 has been documented to be closely linked to the existence and progress of liver fibrosis, triggered by numerous factors [41]. It is noted that STAT3 activation could cross-talk with TGF-β1 signaling in HSCs, exacerbating liver injury. In addition, the knockdown of STAT3 mRNA by siRNA could suppress the expression of TGF-β1 [37]. Many synthetic STAT3 inhibitors have been designed as chemical probes to endorse the impact of STAT3 in chemical-induced liver injury. For instance, STAT3 dimerization inhibitor “STX-0119” is suggested to play a role in controlling the development of CCl4 and TAA-induced liver fibrosis through lessening the activated HSCs [42]. Another synthetic inhibitor, “HJC0123”, has been shown to render the liver more resistant to fibrosis by inhibiting the phosphorylation, nuclear translocation, and transcriptional activity of STAT3 [43]. Moreover, some herbal medicines have shown an inhibitory effect on STAT3 and offered protection against liver fibrosis induced by CCl4, for instance; cucurbitacin-B from many plants of the family Cucurtitaceae [44]; asiatic acid extracted from *Centella asiatica* [45]; S-allyl-cysteine (SAC) from aged garlic extract [46]; and “CCM111” extracted from *Antrodia cinnamomea* [47].

Here, lycorine was able to diminish STAT3 activation, as evidenced by decreasing the phospho-STAT3 (Tyr-705) expression induced by TAA in hepatic tissue, which in turn protected against liver fibrosis. This was corroborated by the lowered antiapoptotic Bcl-2 in conjunction with amplified apoptotic Bax mRNA expressions, leading to the rise of the calculated Bax/Bcl-2 ratio. The observed findings are in accordance with the study of Kang et al. [25], in which lycorine inhibited STAT3 activation in LPS-challenged RAW264.7 cells in-vitro. Furthermore, Wu et al. [17] indicated—by molecular docking—that lycorine inactivates phospho-STAT3 (Tyr-705) by directly binding to its SH2 domain. Accordingly, lycorine stimulated apoptosis in human colorectal cancer cells in-vitro, as evidenced by the activation of caspase and the increase in the ratio of Bax/Bcl-2. In this regard, lycorine is considered to be an apoptosis inducer of both mitochondrial and death receptor-mediated pathways in cancer cells, like breast and bladder cancer, in addition to hematological malignancies, including leukemia and myeloma. It was found that lycorine downregulates the expression of antiapoptotic Bcl-2 family proteins and increases the expression of proapoptotic BAX [48,49]. Recent studies have indicated that the induction of apoptosis in HSCs could ameliorate the progression of liver fibrosis [50,51].

## 4. Materials and Methods

### 4.1. Chemicals

Thioacetamide (TAA) was bought from Sigma-Aldrich (Cat. #: 163678, St. Louis, MO, USA). Lycorine hydrochloride was purchased from Molport^®^ (Compound #: MolPort-009-653-412, Riga Latvia). Neutral buffered formalin solution 10% (Cat #: HT501128) was obtained from Sigma-Aldrich, St. Louis, MO, USA. In addition, phosphate-buffered saline (PBS) was purchased as a powder (pH 7.4) to prepare 1 L solutions (Cat. #: P3813, Sigma-Aldrich, MO, USA).

### 4.2. Animals

Thirty male Wistar rats (age 6–8 weeks, weighing 130–160 g) were purchased from King Fahd Medical Research Center, King Abdulaziz University, Jeddah, and were then acclimatized for seven days at the vivarium of Faculty of Pharmacy, King Abdulaziz University, under a 12 h light/dark cycle, at 40–60% relative humidity and 23 ± 2 °C of air-conditioned atmosphere, and were fed ad libitum with freely available water. The experimental protocol was approved by the Research Ethics Committee, Faculty of Pharmacy, King Abdulaziz University, Jeddah, Saudi Arabia (Reference #: PH-1442-50). All animal experiments conformed with the guidelines of the National Institutes of Health guide for the care and use of Laboratory animals (NIH Publication No. 8023, revised 1978) and complied with the Three Rs, “Replacement, Reduction, and Refinement”, originally proposed by the Universities Federation for Animal Welfare [52].

### 4.3. Experimental Design

Rats were allocated into five groups (six animals per group). Animals in group 1 (control group) received the vehicle (isotonic saline) daily by intraperitoneal (i.p.) injection. The second group was injected with TAA at a dose of 200 mg/kg, i.p., dissolved in saline, three per week for four weeks. Treatment groups 3 and 4 were administered lycorine HCl dissolved in saline (equivalent to doses of 0.5 and 1 mg/kg/d lycorine base, respectively, i.p.), starting 1 h after TAA injection (200 mg/kg, i.p.). Group 5 represented drug-alone animals that were injected only with lycorine (1 mg/kg/d). The selected doses of TAA and lycorine were based on a previous pilot experiment along with earlier literature [16,22]. One day after the last drug treatment, the animals were anesthetized using ketamine-xylazine mixture at doses of 50–5 mg/kg; i.p. [53], and blood samples were collected from the retroorbital plexus for serum isolation. Then, rats were killed by cervical dislocation and their livers were quickly dissected out. Representative hepatic tissues from each animal/group were reserved in 10% neutral formalin (max. 48 h) for histological and immunohistochemical staining. Moreover, extra tissue specimens were kept in an RNAlater storage solution for RNA extraction. The sera and remaining tissues were preserved at −80 °C for later homogenization and biochemical analyses. To isolate the serum samples, blood was allowed to clot in plain tubes for 60 min and then centrifuged at 3000 rpm for 5 min to separate the packed blood cells from the supernatant layer “serum”. Hepatic tissue homogenate was prepared as 10% (*w*/*v*) in ice-cold PBS using a homogenizer (IKA^®^ Ultra-Turrax^®^ Disperser, T 25 digital, Staufen, Germany), followed by centrifugation at 4000 rpm for 5 min to separate the homogenate from the tissues debris. The supernatant was then collected as aliquots and stored at −80 °C until required.

### 4.4. Assessment of Hepatic Fibrosis

#### 4.4.1. Determination of ALT, AST, and Hydroxyproline

The serum activities of the intracellular liver enzymes, alanine aminotransferase (ALT) and aspartate aminotransferase (AST), were assessed using commercial colorimetric kits purchased from Spectrum Diagnostics^®^ (Cat. #: 264001 and 260001, respectively, Al-Obour City, Cairo, Egypt). Hydroxyproline was measured in hepatic homogenates using a specific enzyme linked immunosorbent assay (ELISA) kit (Cat. # MBS726071, MyBioSource, San Diego, CA, USA). The kit adopted the competitive ELISA technique, according to the manufacturers’ instructions.

#### 4.4.2. Histopathological Examination

To evaluate hepatic histopathological abnormalities and the degree of fibrosis, paraffin blocks were first prepared from formalin-fixed tissues and sectioned at 4 µm thickness. Then, the liver sections were stained with standard hematoxylin and eosin (H&E) and were examined by light microscopy. The extent of the collagen deposition was assessed by staining other representative tissue sections with Masson’s Trichrome (MT) and Sirius Red (SR) dyes, following standard procedure [54]. The staining quantitation as area % was performed by image analysis software (ImageJ, 1.48a, NIH, Bethesda, MD, USA). Briefly, the color threshold of each image was adjusted as white with color space (red) and (RGB stack) image type. The function “measurement” was set for area, area fraction, and “limit to threshold”. Then, the image was divided into equal squares using the “Plugin-grid” menu, a series of columns of squares were selected and area % was measured by pressing “m” on the keyboard for each of them.

#### 4.4.3. Immunohistochemical Staining of α Smooth Muscle Actin (αSMA) and Transforming Growth Factor β (TGFβ1)

The immunohistochemical (IHC) technique was utilized to detect αSMA and TGFβ1 expressions in hepatic slices by labeling them with an enzyme that reacts with a suitable substrate to give a colored product. Briefly, liver sections were blocked with bovine serum albumin (BSA; 5% in tris buffered saline), then incubated overnight at 4 °C with the primary rabbit monoclonal antibodies to αSMA and TGFβ1 (Cat. #: ab124964 and ab215715, respectively, Abcam^®^, Cambridge, UK). This was followed by incubation with the biotinylated secondary antibody and subsequently streptavidin-HRP. Sections were then stained with 0.02% diaminobenzidine containing 0.01% H_2_O_2_. Counter staining was done by hematoxylin, and the slides were visualized under a light microscope [55]. IHC quantitation as optical density (OD) was accomplished by image analysis software (ImageJ, 1.48a, NIH, Bethesda, MD, USA). Briefly, each image was adjusted as 640 × 480 size and 8 bit image type. The function “measurement” was calibrated and set for the mean gray value. Then, from the tool bar, a circle denoting an area on the image was selected and OD was measured by pressing “m” on the keyboard.

### 4.5. Assessment of Oxidative Status

Malondialdehyde (MDA) and reduced glutathione (GSH) contents were determined via biochemical kits purchased from Biodiagnostic^®^ (Cat. #: MD2528 and CR2510, respectively, Giza, Egypt). The hepatic superoxide dismutase (SOD) enzymatic activity was assessed based on the method of Sun et al. [56].

### 4.6. Assessment of Inflammatory Status

Assessments of tumor necrosis factor-α (TNF-α) and interleukin-6 (IL-6) concentrations in hepatic homogenate were performed utilizing ELISA kits (Cat. #: BMS622 and BMS625, respectively, Thermo Fisher Scientific^®^, Vienna, Austria). In addition, interleukin-1β (IL-1 β) was measured using an ELISA kit (Cat. # MBS175941), purchased from MyBioSource^®^ (San Diego, CA, USA). All kits implemented the sandwich technique of ELISA, based on the manufacturers’ instructions.

### 4.7. Assessment of STAT3 Pathway

#### 4.7.1. Immunohistochemical Staining of Phospho STAT3 (Y705)

The expression of pSTAT3 in hepatic sections was assessed immunohistochemically by incubation with the primary rabbit monoclonal antibody to pSTAT3 (phospho Y705, Cat. #: ab76315, Abcam^®^, Cambridge, UK), following the same standard procedure previously described in Section 4.4.3.

#### 4.7.2. Reverse Transcriptase–Polymerase Chain Reaction (RT-PCR) Analyses of Bax and Bcl-2 Gene Expressions

Extraction of the total RNA from frozen hepatic tissue was done by RNeasy Mini Kit (Qiagen Inc., Hilden, Germany), as indicated by the manufacturer’s protocol. The extracted RNA was then quantified by spectrophotometry, followed by agarose gel electrophoresis and ethidium bromide to assess integrity. Quantitative assessment of Bax and Bcl2 mRNA expressions was carried out on step one plus (Applied Biosystems^®^, Waltham, MA, USA), using SYBR Green Master Mix, gene-specific forward and reverse primers (10 µM), cDNA, and nuclease-free water. PCR primer sequences are shown in Table 1. Cycling conditions were performed as follows: 10 min at 95 °C, then 40 cycles of 15 s at 95 °C and 60 s at 60 °C. The expression of each assessed genes was normalized relative to that of GAPDH mRNA using the ΔΔ^Ct^ method.

### 4.8. Determination of the Total Protein Content in Hepatic Homogenates

The total protein content in rat liver homogenates was measured according to the manufacturer instructions using a colorimetric BCA protein assay kit (Cat. #23227, Thermo-Fisher Scientific^®^ (Vienna, Austria).

### 4.9. Statistical Analysis

Data are displayed as mean ± standard deviation (S.D.). Statistical analyses were done by one-way analysis of variance (ANOVA), then Tukey’s multiple comparisons were used as a post-hoc test at *p* < 0.05. GraphPad Prism software version 8 (La Jolla, CA, USA) was utilized for both statistical analyses and sketching graphs.

## 5. Conclusions

**Figure 9:**
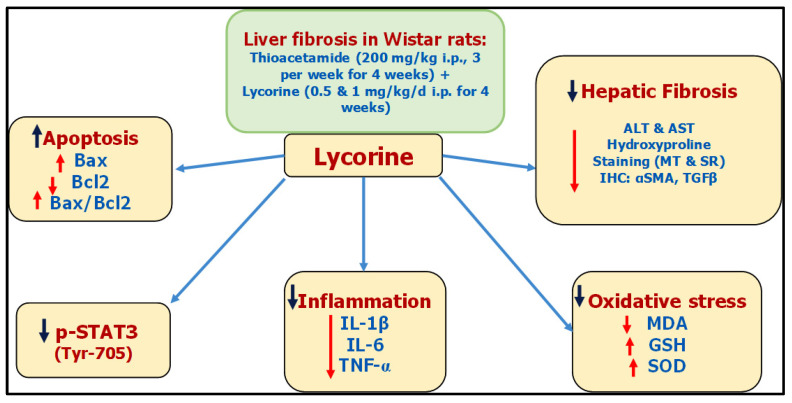
A collective diagram of lycorine antifibrotic effects against TAA-induced hepatic fibrosis in rats.

Based on the present study, it could be concluded that lycorine hinders TAA-induced liver fibrosis in rats, due to—at least partly—its antioxidative and anti-inflammatory properties along with its ability to inhibit STAT3 signaling. These proposed mechanisms have been summarized as a collective diagram, as shown in Figure 9. However, more studies are needed in order to establish the clinical applicability of lycorine treatment in patients with active liver fibrogenesis. The experimental beneficial effects of lycorine in ameliorating liver fibrosis require additional confirmatory studies in the clinical setting to assess its safety and efficacy.

## Figures and Tables

**Table 1:** The sequences of PCR primer pairs used for Bax, Bcl2, and GAPDH.

Primer	Sequence	Gene Bank Association Number
Bax	Forward: 5′-TTCAACTGGGGCCGCGTGG TT-3′ Reverse: 5′-GGAGAGGAGGCCTTCCCAGCCA-3′	XM_011250780.1
Bcl2	Forward: 5′-ATCGCTCTGTGGATGACTGAGTAC-3′ Reverse: 5′-AGAGACAGCCAGGAGAAATCAAAC-3′	XM_0512835.1
GAPDH	Forward: 5′-TCCCTCAAGATTGTCAGCAA-3′ Reverse: 5′-AGATCCACAACGGATACATT-3′	NM_ 028301.2

## Data Availability

Data is contained within the article.
